# Implementing shared care models for young people with mental health difficulties: a consolidated framework for implementation research- informed scoping review of service integration across physical, sexual and mental health domains

**DOI:** 10.1186/s12913-026-14178-x

**Published:** 2026-02-20

**Authors:** Allyson J. Gallant, Karen O’Connor, John Paul Lyne, Leona Ryan, Michelle Doody, Greg Sheaf, Agnes Higgins, David Cotter, Rebecca Murphy, Louise Doyle, David McEvoy, Brian Keogh, Shaakya Anand-Vembar, Mary Cannon, Gary Donohoe, Olivia Longe, Colm McDonald, Sara Burke, Colm Healy, Lorna Staines, David Mongan, Donal O’Keeffe, Caroline Wilson, Yulia Kartalova-O’Doherty, Catherine D. Darker

**Affiliations:** 1 Discipline of Public Health and Primary Care, The University of Ireland Trinity College Dublin, Russell Building, Tallaght Cross, Dublin, D24 DH74 Ireland; 2 Dr. Steevens’ Hospital, Steeven’s Lane, Dublin 8, Dublin, D08 W2A8 Ireland; 3 Department of Psychiatry, Royal College of Surgeons in Ireland, 26 York Street Campus, Dublin, D02 P796 Ireland; 4 School of Psychology, University of Galway, University Road, Galway, H91 TK33 Ireland; 5 Mental Health Ireland, -11- 13 Clarence St, Dún Laoghaire, Dublin, A96 E289 Ireland; 6 School of Nursing and Midwifery, The University of Ireland Trinity College Dublin, The Gas Building, 24 D’Olier Street, Dublin, D02 T283 Ireland; 7 School of Nursing, Psychotherapy and Community Health, Dublin City University, Glasnevin Campus, Dublin, D09 V209 Ireland; 8 School of Medicine, University of Galway, University Road, Galway, H91 TK33 Ireland; 9 School of Medicine, University College Dublin, Health Sciences Centre, Belfield, Dublin 4, Dublin, IE Ireland; 10 Centre for Public Health, Queen’s University Belfast, Belfast, BT12 6BA Northern Ireland UK

**Keywords:** Adolescent, CFIR, Delivery of healthcare, Integrated care models, Youth services, Physical health, Implementation science, Mental health, Review, Sexual health, Policy implementation

## Abstract

**Background:**

Most mental health difficulties (MHD) emerge during adolescence and early adulthood, placing young people at an increased risk for co-occurring physical and sexual health challenges. Shared models of care (SMOC) to connect specialist mental health care with physical and/or sexual health have been developed to address these health needs among young people with MHD. We aimed to identify and characterise SMOC that integrate physical and/or sexual healthcare for young people with MHD, and to synthesise SMOC implementation determinants using the Consolidated Framework for Implementation Research (CFIR) for policy makers, commissioners and practitioners seeking to strengthen youth-integrated service delivery.

**Methods:**

Five electronic databases and key grey literature sites were searched in October 2024. Studies were eligible for inclusion if they predominantly included young people (aged 10–25) with an MHD. SMOC had to address MHD as a primary concern or have parity with the physical and/or sexual health concern(s) being addressed. Key study details were extracted and were appraised using the mixed methods appraisal tool. Screening was conducted in duplicate, with extraction and appraisals conducted by one team member and verified by a second. Findings were thematically synthesised and mapped to CFIR domains to inform implementation planning in youth health systems.

**Results:**

Search results identified 25 relevant SMOC to include in the review. Almost all models (*n* = 23/25) addressed shared care between mental and physical health, while nine addressed mental and sexual health and seven addressed mental, physical and sexual health needs. Reporting quality varied but most SMOC included referral pathways, assessment, treatment and external support components. Barriers frequently mapped to the inner and outer setting CFIR domains, with high staff turnover (*n* = 9) and societal stigma towards mental health (*n* = 7) common concerns. Enablers frequently mapped to the process and innovation constructs, including offering youth-specific care models (*n* = 7) and clear communication between services (*n* = 5).

**Conclusions:**

Despite evidence supporting the need for an integrated care approach, implementation remains limited by setting-specific barriers. Findings highlight the need for service planning and developing tailored, youth-specific models to ensure a holistic approach to care is available to young people experiencing MHD.

**Registration:**

Open Science Framework (osf.io/rj783).

**Supplementary Information:**

The online version contains supplementary material available at 10.1186/s12913-026-14178-x.

**Table:** 

Text box 1. Contributions to the literature	
• Identifies setting-specific barriers (e.g., funding models, staff turnover) and cross-cutting enablers (e.g., youth-tailored design, interprofessional collaboration) to shared models of care (SMOC) across mental health and physical and/or sexual healthcare for young people with mental health difficulties.	
• Highlights a critical gap in the integration of sexual health within youth focused SMOC.	
• Supports health services design through identification of multilevel determinants to inform future implementation efforts.	

## Background

Mental health difficulties (MHD) most often begin during adolescence and early adulthood, with the World Health Organization (WHO) estimating that 50–75% of all MHD emerge before age 25 [1]. MHD encompasses a broad spectrum of conditions, from anxiety and depression to more complex presentations such as psychosis, and are associated with significant long-term consequences when left unaddressed [2]. A recent report from *the Lancet* on the global burden of MHD noted rates of depression and anxiety among those aged 10–24 has grown significantly since 1990, in line with growth of disability-adjusted life years among this cohort worldwide [3]. Additional research from the United Kingdom identified the need for mental health services among children and young people has doubled between 2019 and 2025, with one in five children having a MHD [4].

The health needs of these adolescents and young adults also extend beyond mental health to include their physical health and sexual wellbeing. For example, MHD treatment can contribute to early onset of chronic diseases (e.g., premature cardiovascular disease) in children and adolescents [5, 6]. As onset of MHD can coincide with puberty, the sexual health of young people with MHD is also of concern to support positive health and social development [7, 8]. While sexual health may be considered under the umbrella of physical health, the care needs and service pathways do warrant its own investigation, particularly among young people [7]. The sexual behaviours of young people with MHD are of particular concern as these behaviours have been associated with increased risk for poor health outcomes compared to their peers [9–13]. However, these physical and sexual health domains are frequently overlooked due to MHD diagnostic overshadowing, ambiguous clinical responsibility (e.g., metabolic monitoring), and workforce or service capacity constraints [14, 15]. Despite increasing policy attention, young people with MHD often experience fragmented, siloed, or inaccessible primary or secondary healthcare services [16–20].

Shared models of care (SMOC)— coordinated care across primary care (e.g., general practitioners [GP]) and secondary care (e.g., specialist mental health services) to support patient referrals, diagnoses, treatment and monitoring, and discharge planning —offer a promising strategy to deliver more holistic, youth-centred services to address physical, sexual and mental health concerns [21, 22]. International evidence highlights SMOC are effective at improving patients’ engagement and treatment with mental health care, while increasing the accessibility of high-quality healthcare [22–24]. Healthcare providers have acknowledged the importance of this approach to integrated care, yet it remains inconsistently implemented and rarely evaluated [22–25]. Understanding how health system structures enable or constrain implementation of SMOC is critical for effective and equitable service delivery.

Implementation science provides a means to address this gap by shifting attention from intervention efficacy to the contextual factors that shape real-world uptake and maintenance [26]. Implementation of SMOC targeting young people is particularly complex given the cross-sectoral nature of services and the need for coordinated roles, communication, and accountability [27]. Frameworks such as the Consolidated Framework for Implementation Research (CFIR) support the systematic identification of implementation determinants, and has been widely applied to youth-focused health services research to identify barriers and enablers to innovation implementation [26, 28–30]. Existing evidence syntheses have sought to address concepts regarding young people’s MHD, SMOC, and physical and sexual health concerns among those with MHD [31–34]; however, there has yet to be a review which has synthesized SMOC across mental, physical and/or sexual health among young people with MHD. Therefore, we sought to (1) identify and describe SMOC that address the physical and/or sexual health of young people with MHD, and (2) synthesise the implementation barriers, enablers, and determinants of these models using CFIR. Review findings can directly address youth mental health policy and the service delivery and implementation challenges central to health system reform.

## Methods

### Protocol & registration

The review protocol was registered with open science framework (osf.io/rj783) and previously published [35], with any deviations from the protocol described below. The review was conducted using JBI scoping review methodology and reported according to Preferred Reporting Items for Systematic Reviews and Meta-Analyses- Extension for Scoping Reviews (PRISMA-ScR) checklist [36, 37]. JBI’s Population–Concept–Context (PCC) framework underpinned the methods, as described below [37].

### Conceptual framework

CFIR is a comprehensive framework that consolidates constructs known to influence implementation across five domains: innovation (e.g., an intervention, program, policy being implemented), characteristics of individuals (e.g., those involved in planning, providing or receiving the innovation), inner setting (e.g., the setting[s] where the innovation is delivered), outer setting (e.g., the broader setting[s] in which the inner setting[s] exist), and process (e.g., strategies used to implement the innovation) [26]. We selected CFIR to enable a theory-informed synthesis of the literature and to structure the identification and analysis of implementation determinants, and it is well suited to understanding complex, cross-sectoral research areas such as SMOC [30].

### Searches

A health sciences librarian (GS) developed the electronic database search strategy with input from the research team to identify relevant peer-reviewed literature. The team first defined search terms aligned with the review’s population (i.e., young people), concept (i.e., mental health, physical and/or sexual health), and context (i.e., shared care across primary and secondary settings.) The librarian then developed initial search strings for Web of Science (Core Collection), run on 21 October 2024. These were subsequently adapted for MEDLINE (EBSCOhost), Embase (Elsevier), PsycINFO (EBSCOhost), and CINAHL (EBSCOhost) and applied between 25–29th October 2024 (Additional file 1). No date, language, or geographical restrictions were applied. Search results were uploaded to Covidence (Veritas Health Innovation, Melbourne, Australia) for de-duplication and screening.

### Grey literature

We searched the websites of national health organisations and think tanks to identify relevant grey literature on 31 October 2024. Key terms (e.g. mental health, sexual health, shared care) were used in each website’s search function and results were screened until 25 consecutive irrelevant links were reached. Reference chaining was used within identified documents. Audio-visual materials (e.g. webinars, podcasts) were excluded.

### Study inclusion and exclusion criteria

Eligibility criteria included studies focused on young people aged 10–25 years with MHD [2, 38]. Studies including broader age ranges were eligible if at least 60% of participants were within the target age range, or if data could be disaggregated. Studies focusing on youth without MHD, those solely with substance use disorders, or adults aged ≥ 26 were excluded.

MHD encompassed “*the full range of mental health difficulties that might be encountered, from the psychological distress experienced by many people to severe mental disorders that affect a smaller population*“ [2]. MHD included in the International Classification of Diseases- 11th Revision (ICD-11) which affect young people were eligible for inclusion [39]. Neurodevelopmental conditions as classified by the ICD-11 (e.g., intellectual disabilities, autism spectrum disorder) were considered when they were coupled with another MHD [39]. Physical health was defined as the “*condition of your body, taking into consideration everything from the absence of disease to fitness level, ”* and sexual health as “*a state of physical, emotional, mental and social well-being in relation to sexuality; it is not merely the absence of disease, dysfunction or infirmity. Sexual health requires a positive and respectful approach to sexuality and sexual relationships, as well as the possibility of having pleasurable and safe sexual experiences, free of coercion, discrimination and violence*” [40, 41]. There were no restrictions placed on physical or sexual health presentations included in the review.

SMOC were included when MHD were the primary concern or given equal weight to physical and/or sexual health concerns. Studies focusing solely on MHD, physical or sexual health concerns were excluded, as were studies focusing on well-child visits. Included studies had to describe shared care between primary and secondary settings. Eligible settings included healthcare clinics, hospitals, school or university health services, sexual health clinics, and other community-based or non-traditional settings. Virtual shared care (e.g., teleconsultation) was eligible, but standalone digital interventions (e.g., apps) were excluded. Studies describing internal quality improvement within primary care, without external mental health service involvement, were also excluded.

All empirical study designs were eligible, including quantitative, qualitative, and mixed methods designs, as well as relevant theses, dissertations, and grey literature. Protocols, opinion pieces, book chapters, conference abstracts, and other reviews were excluded. Reference chaining from relevant reviews was conducted to identify additional eligible studies.

### Screening

Two independent reviewers (AJG, CDD) screened 10 titles and abstracts to pilot test and calibrate against the inclusion criteria. After piloting, all remaining citations were screened in duplicate. Full-texts of potentially eligible articles were uploaded to Covidence and screened again in duplicate. Reasons for exclusion at the full-text stage were recorded and are detailed in the PRISMA-ScR flow diagram. Conflicts at each stage were resolved through discussions between reviewers. When eligibility was unclear, the corresponding author was contacted. Nine authors were contacted with two responding, resulting in the inclusion of one article.

### Data extraction

Included studies underwent data extraction. The initial template included descriptive study details (e.g., authors and year of publication, country, study design, aim), population details (e.g., characteristics of young people, mental health presentation, sample size), concept (e.g., physical health and/or sexual wellbeing concerns), context (e.g., description of SMOC, settings) and determinants (e.g., barriers, enablers and implementation determinants). This template was pilot tested by the two reviewers with three included studies. This resulted in several modifications to the template including: term(s) to describe SMOC; healthcare personnel involved in the model of care; any theory, model or framework underpinning the SMOC or research study; and study outcomes. The updated template was tested with two further included studies to ensure all relevant details were captured, with no further edits made during this process. The reviewer (AJG) continued data extraction for all remaining studies which were verified by a second team member (LR, CDD). Any concerns from the verifier were flagged for the lead reviewer to revise.

### Study quality assessment

Critical appraisals were conducted using the mixed methods appraisal tool (MMAT) to assess included quantitative, qualitative and mixed methods studies [36, 42]. The MMAT has five appraisal criterions for each study design, with responses coded as ‘yes’, ‘no’ or ‘can’t tell’, with ‘yes’ results summed [42]. Two reviewers (AJG, CDD) piloted the tool with three studies of differing designs, with AJG completing appraisals thereafter, with work verified by LR or CDD. All studies were retained regardless of MMAT score.

### Data synthesis and presentation

Extracted data were aggregated and summarised to address review objectives and presented using narrative summaries, tables and figures, where appropriate. Descriptions of included studies were also compiled into a table to support the narrative. We used reflexive thematic analysis to synthesise descriptive SMOC characteristics and implementation features [43]. Following an initial review of the included studies, two team members (AJD and CDD) derived key codes and subcodes from the extracted data, which reflected critical SMOC components and their implementation. Developed codes and subcodes were then categorised into a thematic framework and presented narratively. Barriers and enablers to SMOC interventions and their implementation determinants were mapped to the five high-level CFIR domains and summarised narratively, with supporting tables and figures.

## Results

### Review statistics

**Fig. 1:**
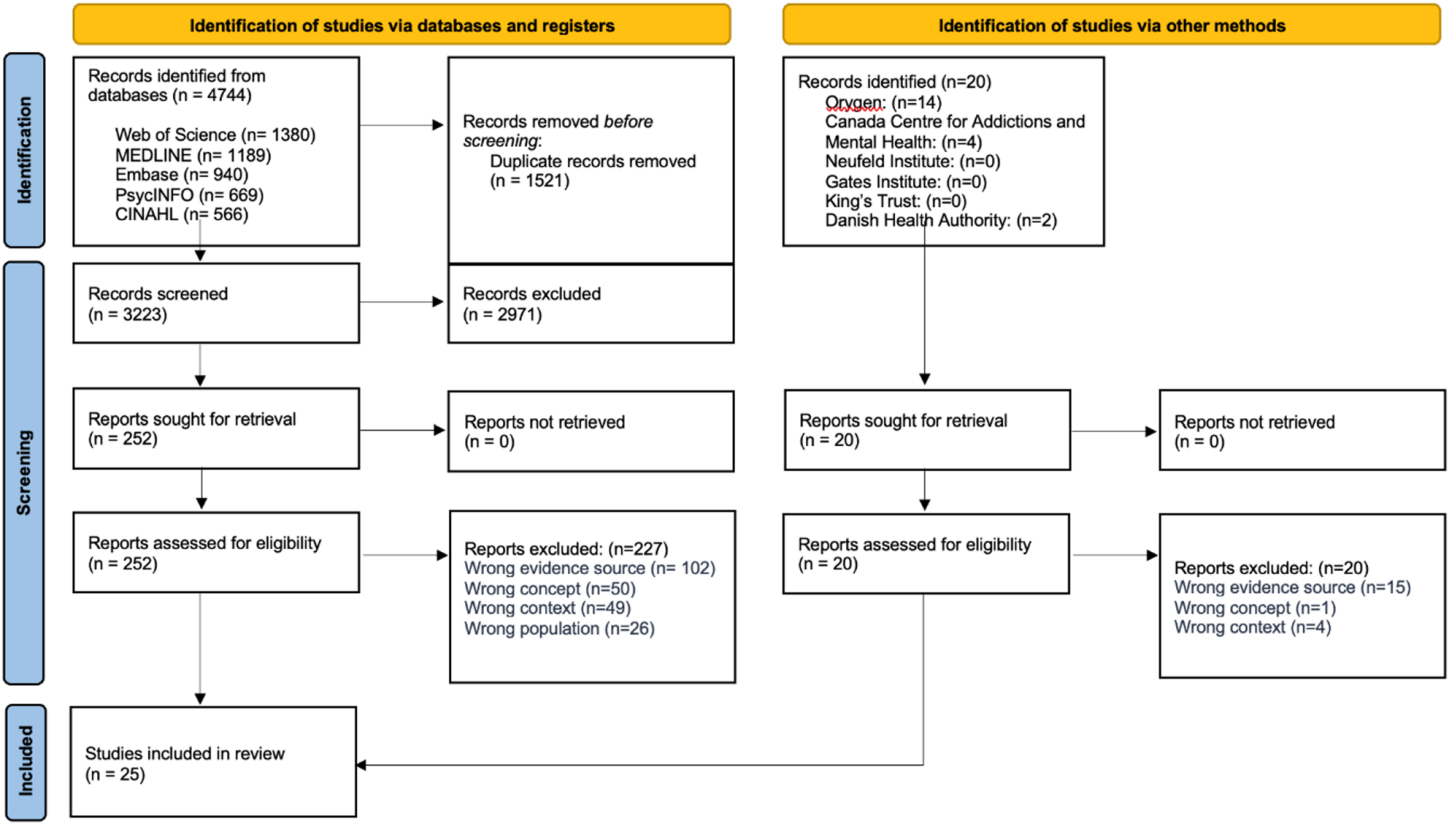
PRISMA-ScR Flow Diagram [36]

Database searches yielded 4,744 records, of which 3,223 were screened at the title and abstract level after de-duplication. A total of 252 full- text articles were screened, with the majority excluded due to ineligible evidence source (*n* = 102), concept (*n* = 50), context (*n* = 49) or population (*n* = 29). No additional eligible studies were identified through grey literature searches. In total, 25 studies met inclusion criteria and were included in the final review. The screening process is detailed in the PRISMA-ScR flow diagram (Fig. 1).

### Description of included studies

**Fig. 2:**
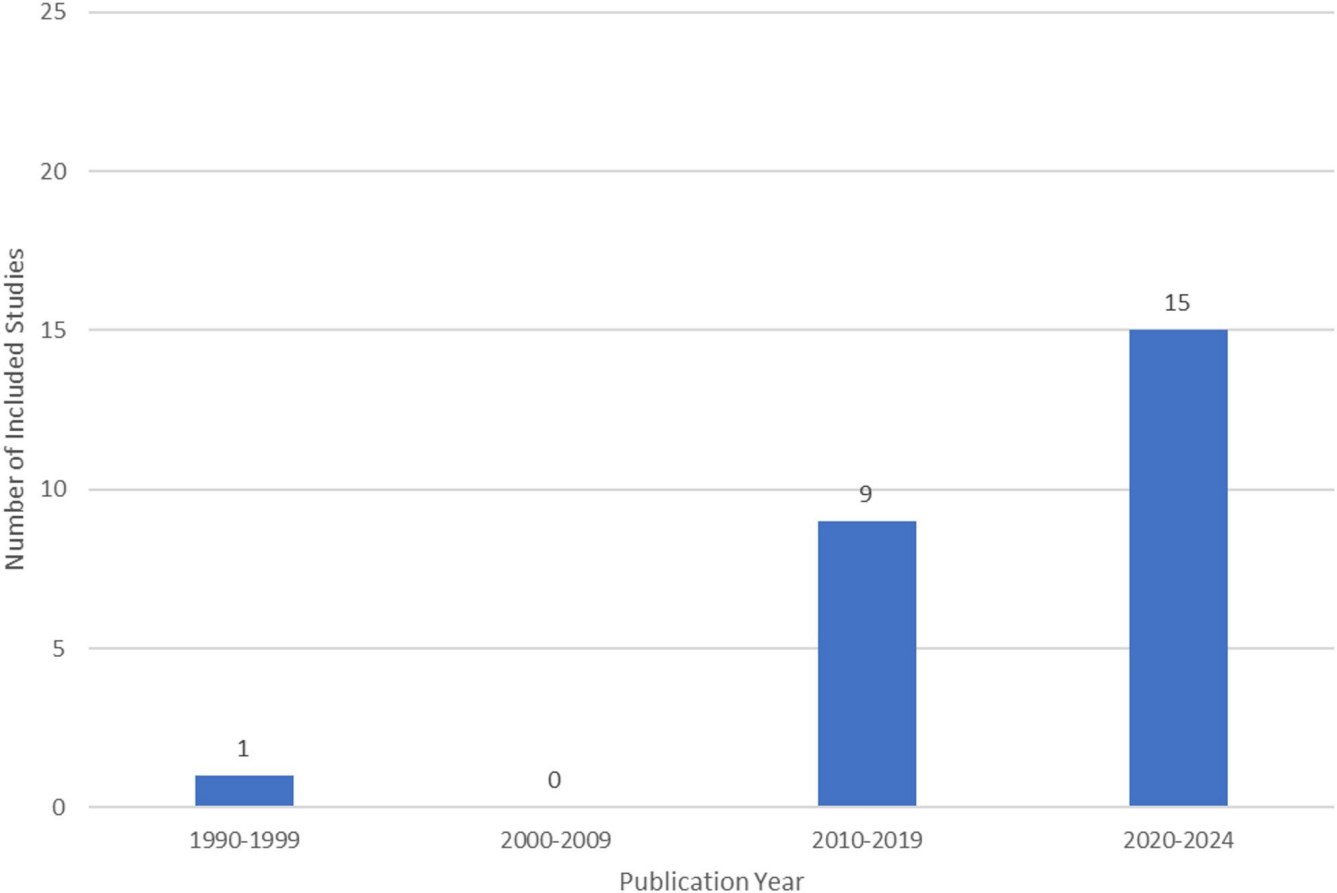
Publication year of included studies

Table 1 below summarises the characteristics of included studies. The majority of included studies were conducted in high-income countries, most commonly the United States (US; *n* = 15) and Australia (*n* = 5). Only one study was from a low- or middle-income country (Mozambique) [44]. Publication years ranged from 1993 to 2024, with the majority (*n* = 16) published since 2020 (Fig. 2). Study designs included quantitative (*n* = 10), qualitative (*n* = 9) and narrative (e.g., describing SMOC development; *n* = 6) approaches.

**Table 1:** _Details of included studies

Lead author surname & year	Country	Study objective	Study design	Study population	Targeted population of shared model of care and any noted mental health difficulty/ies	Physical health concern(s)	Sexual health concern(s)	Shared Model of Care description	Implementation of shared model of care	Primary and secondary care settings included in shared model of care	Outer setting or context
Adams et al., 2016	US	To highlight an integrated care model which includes school psychologist trainees and medical residents to collaborate on integrated care across schools and primary care settings.	Narrative description	Youth (*n* = 60); parents (*n* = 11); pediatricians (*n* = 8)	General behavioural health needs (e.g., noncompliant behaviour, tantrums, mood challenges)	Weight check, diet, medication monitoring	None reported	Integrating school psychology trainees into behavioural health services to improve comprehensive care, early MHD intervention and accessible MHD care.	None reported	Schools and primary care clinics	Education and health systems/ sectors
Brunette et al., 2023	US	To provide baseline data for patients using the prohealth new hampshire integrated care program.	Descriptive quantitative study	Young people aged 16–35 with serious MHD (*n* = 122)	Serious MHD, including schizophrenia, bipolar disorder, major depression, post-traumatic stress disorder and anxiety disorder.	Body mass index, blood pressure, (pre)diabetes screening, cardiovascular disease and other chronic health conditions, medications	None reported	Prohealth New Hampshire: youth receive health screenings in primary care and care is coordinated with mental health services. Youth receive tailored lifestyle programming when needed.	None reported	Primary care clinics and community mental health clinics	Federally qualified health centers, American insurance policies
Eapen et al., 2022	Australia	To outline how the integrated continuum of connect and care model was developed.	Narrative description	Young people aged 12–17 with MHD	General MHD (discussed generally)	Physical health (discussed generally)	None reported	Integrated continuum of connect and care model: seeks to offer integrated care and timely access to mental health care for young people. In-person or digital assessments of young people are first conducted and then linked to relevant care needs along a tiered care pathway.	None reported	Model to be implemented across health settings, education, social services, disability support, out of home care, nongovernment services, early learning/ education providers, priority populations including the aboriginal and out of home care services	Australian government department of health
Feingold & Slammon, 1993	US	To present a model of integrated care for mental and medical health service delivery across hospital and community-based primary care.	Narrative	Families of children with HIV or suspected to have HIV	MHD (discussed generally)	None reported	HIV/AIDS	The No One Along with AIDS pathway: Designed to support integration of primary care and community mental health service needs of children with HIV.	None reported	Boston city hospital and the three participating neighbourhood health centres	Conducted during HIV/ AIDS epidemic
Hacker et al., 2013	US	To explore how pediatricians incorporate behavioural health screening in a setting where behavioural health screening and associated mental health referrals.	Qualitative	Primary and secondary care providers (*n* = 14)	Youth (aged 6–18 years) with behavioural and MHD (e.g., anxiety, depression, and nonspecific problems)	Medication monitoring	None reported	Mandated behavioural health screenings in pediatric primary care with appropriate referrals to co-located mental health services.	None reported	Pediatric primary care and co-located child and adult psychiatry services	Insurance, provider compensation/finances
Henderson et al., 2023	Canada	To describe the development of youth wellness hubs.	Narrative	Youth aged 12–25 with mental health needs	General MHD (generally described)	Medication monitoring	Sexual health (general)	Integrated youth services: comprehensive primary care program with links child and adolescent mental health services, including community and hospital-based services, in addition to comprehensive (e.g., housing and education) needs.	None reported	Adolescent and adult mental health and substance use services, as well as services from across other youth sectors (e.g., housing, education).	Ontario ministry of health
Hine et al., 2017	US	To identify physician’s perspectives on the benefits of having a pediatric behavioural health practitioner included in integrated care.	Cross-sectional survey	Pediatricians (*n* = 27)	Youth and families with behavioural health needs (generally described)	Physical health follow-up generally described	None reported	Patient was referred by a pediatrician to on-site behavioural health services. Warm hand-offs and communication channels also highlighted (e.g., progress notes, in-session and hallway consultations.)	None reported	Integrated behavioural health centres and primary care.	University of nebraska medical center state’s academic health sciences center
Hugunin et al., 2023	US	To explore pediatricians and psychiatrists’ perspectives of shared care for young people with mental health difficulties.	Qualitative	Pediatricians and psychologists (*n* = 21)	Young people with serious mental health difficulties (generally described)	Medication and side effect monitoring	None reported	Integrated care models discussed generally, but no specific model outlined.	Ideas for processes mentioned but none were implemented	Primary and secondary care settings	Mental health stigma, insurance policies, siloed health and insurance systems
Katz-Wise et al., 2020	US	To explore clinic staff’s perspectives to providing sexual and reproductive health care to young women with depressions.	Qualitative	Young women with depression, healthcare providers in primary care and sexual health clinics (*n* = 28)	Young women with depression	None reported	Sexual and reproductive health (e.g., contraception, condom use, sexually transmitted infections and unintended pregnancies)	Providing comprehensive sexual and reproductive health care to depressed young women through work with sexual health clinics and primary care.	Study’s goal was to determine process factors and considerations for service delivery	Primary care and sexual health clinics	Patient laws, health insurance policies
Lai et al., 2016	US	To describe the first year of a school-based wellness center network and identify the barriers and enablers to the care centers.	Qualitative	Staff involved with school-based health centres (*n* = 43)	School-aged children with MHD (depression and others generally described)	Physical health (generally described), diabetes	None reported	School-based health centres, which offer primary care and mental health services. Some settings also offer comprehensive health services (e.g., vision and dental care.)	None reported	Schools, local health system	Federally qualified health centers, American insurance policies, school districts
Liu et al., 2010	US	To identify characteristics of a school-based health centre and its ability to integrate mental health services and address the mental health needs of youth.	Cross-sectional survey	School based health centre staff (*n* = 42) and patients (*n* = 590)	School-aged children with mental health difficulties. Designed for general presentations but specific difficulties mentioned included aggressive or disruptive behavioural issues, anxiety, stress, mood disorders.	Physical health (generally described)	None reported	School-based health centres, which offer primary care and mental health services in varying levels of integration. At minimum, schools need to include mental health screening and assessments, with referrals to additional services as needed.	None reported	Schools with school-based health centres	Oregon state program office which funds the program.
Lovero et al., 2022	Mozambique	To describe the use of implementation mapping and workshops to design a shared model of care to treat adolescents with depression.	Narrative	Staff within primary care clinics, adolescents with depression (*n* = 29)	Young people aged 12–19 with depression	Physical health (generally described)	Sexual health (generally described)	Young people could be screened for mental health concerns at their entry-point to care, with positive screens referred to same-day mental health care (e.g., group therapy sessions.)	Workshops used to identify implementation strategies	Offices of mental health and school and youth health, selected primary care clinics	Ministry of health, departments of school and youth health, and primary health care, the Mozambican national health system, ministry of health, department of mental health at the Mozambican ministry of health, national mental health program.
Lyon et al., 2016	US	To explore the potential usefulness of collaborative care in school-based mental health services.	Narrative	Health and education staff	Mental, behavioural and/or emotional concerns (generally described)	Physical health concerns (generally described), medication monitoring	None reported	ACCESS model: designed to enhance coordination of school mental health service delivery and additional support services (both internal in the school and with external community services).	Lots of discussion of education/training, communication	Schools and primary care clinics	Broader health and education sectors
Mann et al., 2021	US	To determine how healthcare system use varies among children with mental health difficulties following the implementation of a shared approach to care.	Retrospective chart audit	Youth with emotional and mental health conditions (*n* = 15)	Youth under age 18 with complex mental and behaviour difficulties in general. Specific conditions mentioned included: generalized anxiety disorder, major depressive disorder, post-traumatic stress disorder; conduct disorders, bipolar and disruptive mood disorders.	Physical health concerns (generally described). Examples of specific conditions mentioned included: abdominal pain, pharyngitis, fever, musculo‑ skeletal complaints.	None reported	Shared plans of care	None reported	Division of child and community health’s regional centers in Iowa	Health sector
Mathias et al., 2022	Canada	To summarize preliminary findings from a comprehensive, integrated care plan, including qualities of the services provided and patients accessing the services.	Narrative with retrospective chart review	Youth aged 12–24 (*n* = 4783)	mental health (generally described)	physical health (generally described)	sexual health (generally described)	Foundry services: designed to offer seamlessly integrated care for young people with mental health difficulties, including physical health care, sexual health, mental health, substance use, youth support or other social services.	None reported	The six physical foundry centres and online resources	Health and education sectors, funding
Mautone et al., 2021	US	To describe program development, implementation and evaluation of a large-scale integrated care model.	Narrative with cross-sectional survey	Primary care, psychiatry department leadership, psychologists, psychiatrists (*n* = 60)	Young people with existing MHD. Examples provided included disruptive behavior (including tantrums), school problems (including bullying), and mood problems.	physical health generally, sleep problems, adherence to asthma treatment plans.	None reported	Healthy minds, healthy kids: a single setting approach designed to address staff shortages and other care barriers. Behavioural health champions designed to work with primary care to ensure primary care and mental health needs addressed in a comprehensive approach in line with existing workflow and organisation procedures.	A steering committee of primary care network and psychiatry department leadership	The five pediatric primary care practices where model was implemented	The 30-practice network owned by a children’s hospital, insurance plans, electronic medical record software
Morgan et al., 2018	New Zealand	To explore staff’s perceptions about the complexities of collaborative care for young people with MHD.	Qualitative	Staff involved in care delivery (*n* = 20)	Youth with complex MHD (generally described)	Physical health (generally described)	Sexual and reproductive health, contraception	Wraparound care for youth which includes community-based, integrated, youth-friendly health and social services. Can link to additional appropriate culturally appropriate care and external community supports (e.g., employment, housing).	None reported	Care clinics, education agencies, child welfare, mental health agencies, social services, justice agencies, mental health crises supports	Broader New Zealand health, education and social sectors
Nash et al., 2020	Australia	To explore clinicians’ experiences of Head Space Early InterventionTeams two years after service implementation.	Qualitative	Healthcare providers involved in care provision (*n* = 9)	Youth aged 14–25 with existing, emerging or apparent MHD. Examples provided include depression, anxiety, schizophrenia and other primary psychotic disorders.	Diet and exercise support	Sexual health (generally described)	Head Space Early Intervention Teams: Model is youth-friendly and co-located to improve person-centred approach to care. Seeks to deliver integrated healthcare, while also offering links to educational, employment and social services. physical health concerns.	None reported	Headspace clinics, primary care centres	Local health districts, Australian government funded the local primary health networks.
Rodriguez et al., 2019	US	To examine providers’ perspectives on a collaborative psychiatry consultation program in pediatric primary care.	Qualitative	Primary and secondary care providers (*n* = 14)	Children and adolescents with MHD (generally described)	Medication monitoring	None reported	Integrated, interdisciplinary care: behavioural health specialists are integrated into a co-located primary care centre to work together with primary care providers to address young people’s behavioural health needs.	None reported	Federally qualified health center	None reported
Rooney et al., 2021	Ireland	To explore the feasibility and identify the perceived barriers and enablers of developing an the extension for community health care outcomes program for child and adolescent mental health services in Ireland.	Qualitative	Consultants, GPs and other primary healthcare professionals (*n* = 29)	child and youth with mental health needs. Examples of specific conditions included anxiety, depression, eating disorders, neurodevelopmental delays and trauma disorders.	Psychotropic medication management	None reported	Child and Adolescent Mental Health Services: using virtual consultations to extend the current services available to young people with mental health difficulties in Ireland while addressing staff shortages and other barriers to care.	GP upskilling through training and workshops	Telehealth service to connect stakeholders	Health service executive
Rungan et al., 2024a	Australia	To describe partnerships between the health and education sector within school-based health centres.	Qualitative, as part of a broader a critical realist-informed three-phase, sequential mixed method study.	Staff across health and education system (*n* = 7)	School-aged children (aged 5–18) with mental or behavioural difficulties (generally described)	Physical health concerns (generally described)	Sexually transmitted infections	School-based integrated care model: limited reporting on actual shared care, with a broader focus on barriers and facilitators of care model.	None reported	Schools, health clinics	Health, education and social work sectors, Australian indigenous culture
Rungan et al., 2024b	Australia	This provides a comprehensive description of the Kalgal Burnbona framework development and implementation.	Narrative	Staff across health and education and social welfare system	School-aged children (aged 5–18) with behavioural difficulties (generally described)	Physical health concerns (generally described)	None reported	Kalgal Burnbona: designed to offer youth-focused, inclusive and culturally-appropriate care with co-located services as a way to address young people’s needs while minimizing existing barriers. Models also refer youth to external services, such as education and social services when needed.	Committee development with key stakeholders and indigenous community leaders, financing, training	School-based heath centres which offer mental and primary care.	Broader Sydney local health district
Schweitzer et al., 2023	US	To describe a model of care and cross-sectional implementation satisfaction surveys by primary care providers.	Narrative and cross-sectional survey	Primary care providers, secondary care providers, primary care leads, care coordinators (*n* = 30)	Young people with an identified mild to moderate MHD. Examples provided include traumatic events, challenging behaviors, externalizing behaviours	Medication monitoring	None reported	Primary care mental health integration: designed to improve early mental health intervention, improve communication across sectors and using evidence-informed care across primary care providers and mental health specialists.	Training	Hospital-affiliated primary care practices in San Diego and riverside counties	Leadership commitment, communication
Walter et al., 2018	US	To report findings from a psychiatric consultation program to support primary care providers’ skills in the area.	Observation study and survey	Primary care providers (*n* = 66 survey participants, 81 participants in care model)	Pediatric patients with mild to moderate anxiety, depression or behavioural disorders.	Medication prescribing and monitoring	None reported	Psychiatric consultations offered by child and adolescent psychiatrists offered during weekdays to primary care providers.	Education sessions to support upskilling	41 pediatric practices enrolled in a statewide, independent association of 90 pediatric practices affiliated with an academic medical center	Health system
White et al., 2021	Australia	To describe the patients’ pathway through headspace early intervention teams.	Retrospective chart audit	Youth (*n* = 26)	Youth with MHD (emerging, apparent, or diagnosed.)	Diet and exercise support	Sexual health (generally described)	Head Space Early Intervention Teams: Designed to address gap in specialist mental health and primary care services. Model is youth-friendly and co-located to improve person-centred approach to care. Seeks to deliver integrated healthcare, while also offering links to educational, employment and social services. physical health concerns.	None listed	Primary care and psychiatry clinics	Sydney local health district, Primary health networks

### Study quality assessment

MMAT appraisal results are presented in Additional File 2. Given the included study designs, only the qualitative and quantitative descriptive criteria were used to assess studies. Of the nine qualitative studies, seven met all five scoring criteria [44–50]. One study met four criteria due to limited reporting on data collection [51], while another met three criteria for providing only a narrative summary of workshop themes without supporting quotes [52]. Among the ten quantitative descriptive studies, one met all five MMAT criteria [53]. Most of these studies met four of the five criteria as they did not meet the non-response bias criterion [44, 54–61]. One study met three criteria due to limited detail on data analysis methods [60].

### Description of SMOC

Terms used to describe SMOC varied across studies, including “*integrated behavioural health*” [54, 56], *“collaborative behavioural healthcare”* [53] and “*integrated school-based mental health”* [62]. Six models also provided linkages to other services (e.g., social, vocational, income, housing and/or education) to support young people’s overall wellbeing [48, 49, 58, 61, 63, 64]. Five studies used theories, models or frameworks to inform SMOC development, including the Exploration, Preparation, Implementation, Sustainment framework and the Proctor’s Implementation Outcomes Framework [44, 51, 58, 59, 62].

SMOC also varied in the MHD they targeted. Nine addressed general MHD [48, 50, 52, 57, 58, 64–67], while five focused on complex behavioural and MHD (e.g., anxiety, oppositional defiant disorder) [45, 54, 59, 62, 68]. Four models included complex MHD (e.g., psychosis, schizophrenia) [46, 49, 55, 61], while three included mild to moderate MHD and behavioural health difficulties (e.g., anxiety, anger management) [51, 53, 60], two focused on behavioural needs more broadly [56, 67] and two specifically on depression [44, 47]. Support systems were also integrated into several SMOC, such as including parents (*n* = 6), teachers (*n* = 4) or peers (*n* = 1) [44, 49, 51, 54, 59, 61, 62, 64–66, 68, 69].

Almost all models (*n* = 23) included physical health concerns within their SMOC. Thirteen models generally described addressing the physical health of young patients [44, 48, 50, 51, 56–59, 62, 64, 65, 68, 69], while 10 models described medication monitoring [45, 46, 52–55, 60, 62, 66, 67]. Other physical health concerns identified included addressing diet (*n* = 3) [49, 54, 61] diabetes and/or pre-diabetes (*n* = 2) [50, 55], weight management or body mass index (BMI; *n* = 2) [54, 55], exercise (*n* = 2) [49, 61] and sleep difficulty (*n* = 2) [59, 60]. By comparison, only nine models explicitly incorporated sexual health and wellbeing [44, 47–49, 51, 58, 61, 65, 66]. General sexual health concerns were mentioned in six models [44, 48, 49, 58, 61, 66], with sexually transmitted infections (STIs; inclusive of HIV and AIDS) identified in four studies [47, 48, 51, 65], and two incorporating the promotion of safe sex practices (e.g., contraception, condom use) [47, 48]. Seven SMOC addressed both physical and sexual health [44, 48, 49, 51, 58, 61, 63].

Healthcare personnel from primary care commonly included GPs (*n* = 20) [44, 47, 48, 50–53, 55, 57, 58, 60–62, 64–69] and paediatricians (*n* = 7) [45, 46, 51, 54, 56, 64, 69]. Nurses (*n* = 4) [47, 48, 52, 65], allied health professionals (e.g., dietitians; *n* = 3) [52, 61, 69], nurse practitioners (*n* = 2) [47, 56], physician assistants (*n* = 2) [47, 68] and medical trainees (*n* = 2) [54, 67] were also identified. Three studies specified primary care leadership were involved in the SMOC [59, 60, 69], and two studies included providers with sexual health expertise (e.g., HIV care providers, family planning counsellors) [47, 65].

Psychiatrists were the most common provider involved from secondary care (*n* = 15) [45–47, 49, 52, 53, 59–62, 66–69], with child and adolescent psychiatrists specified in two studies [52, 53]. Social workers (*n* = 12) [45, 48, 51, 54, 57, 59, 60, 62, 65, 67–69], mental health specialists (*n* = 11) [44, 45, 48, 52, 55, 57, 58, 61, 62, 64, 68], psychologists (*n* = 9) [52, 54, 57, 59, 61, 62, 64, 65, 68], mental health trainees (e.g., interns, doctoral candidates; *n* = 4) [49, 54, 56, 67] and psychiatric nurses (*n* = 2) were also included [57, 65].

The most common SMOC setting was primary care (*n* = 15) [44–47, 49, 53–56, 59, 61, 62, 64, 66, 68], with three of these studies specifying paediatric practices [45, 54, 56]. Care was also provided through hospitals (*n* = 7) [46, 53, 59, 64–66], community health centres (*n* = 7) [44, 45, 48, 49, 58, 61, 65], school-based health centres (*n* = 6) [50, 51, 54, 57, 62, 69], community mental and/or behavioural health clinics [55, 60, 66] and a sexual health clinic [47]. Twelve studies provided descriptions of the broader health systems [46–54, 58, 59, 61, 62, 68], and the local government structures (e.g., Sydney’s Local Health District, Ontario Ministry of Health; *n* = 7) SMOC were implemented within [44, 49, 61, 63, 64, 68, 69]. The education sector also was highlighted in seven studies [48, 50, 54, 56–58, 70], with insurers mentioned in six studies, particularly among US studies [45–47, 50, 55, 59]. One model was specifically nested within Australia’s Indigenous community [70].

The reporting and description of SMOC varied across studies but frequently included: referral pathway into shared care; screening and assessment; treatment plans; and referrals to external community services.

Care pathways typically involved referrals by GPs [44, 54, 59, 65], mental healthcare providers [61, 67], or broader members of the patient’s care team [56, 60, 69]. Two models allowed for young people to self-refer for treatment [45, 66]. Mental health screening and assessments occurred early in the process [44, 45, 50, 55, 57, 59, 62, 69], with four SMOC specifying mental health diagnoses or diagnostic clarifications occurred at this stage [57, 61, 62, 68].

Treatment plans were often co-developed with young people and/or their parents (e.g., parents, caregivers or legal guardians; *n* = 12) [44, 48–50, 58, 60, 62, 64–66, 68, 69]. Treatment plans focussed on addressing MHD and were often offered for a finite period (e.g., 8–10 therapy sessions, group therapy for up to 6 months) [44, 48, 49, 54, 55, 57, 59–62, 65, 68, 69]. Four models incorporated patient education [44, 58, 60, 64], flexible appointment times [44] or lifestyle interventions, such as smoking cessation [55].

Nine models included referrals to additional health settings (e.g., emergency departments, in-patient care) for patients, particularly for those with complex MHD [49, 50, 57, 59–62, 64, 65]. Patient follow-up and/or monitoring (e.g., continuing brief cognitive behavioural health interventions, monitoring changes in patient health outcomes) was detailed in eight models [44, 54, 57, 59, 60, 64, 68, 69].

Implementation processes and sustainment were generally under-described. Staff training and education was described in four studies [53, 60, 62, 69]. Training could include support for updated technologies to be used in the model of care, while examples of education modules included the benefits of integrated care or details of psychotropic medications [53, 60]. Ongoing supervision, monitoring and feedback mechanisms were described in two studies [44, 59].

### Barriers, enablers, and determinants mapped to CFIR

**Fig. 3:**
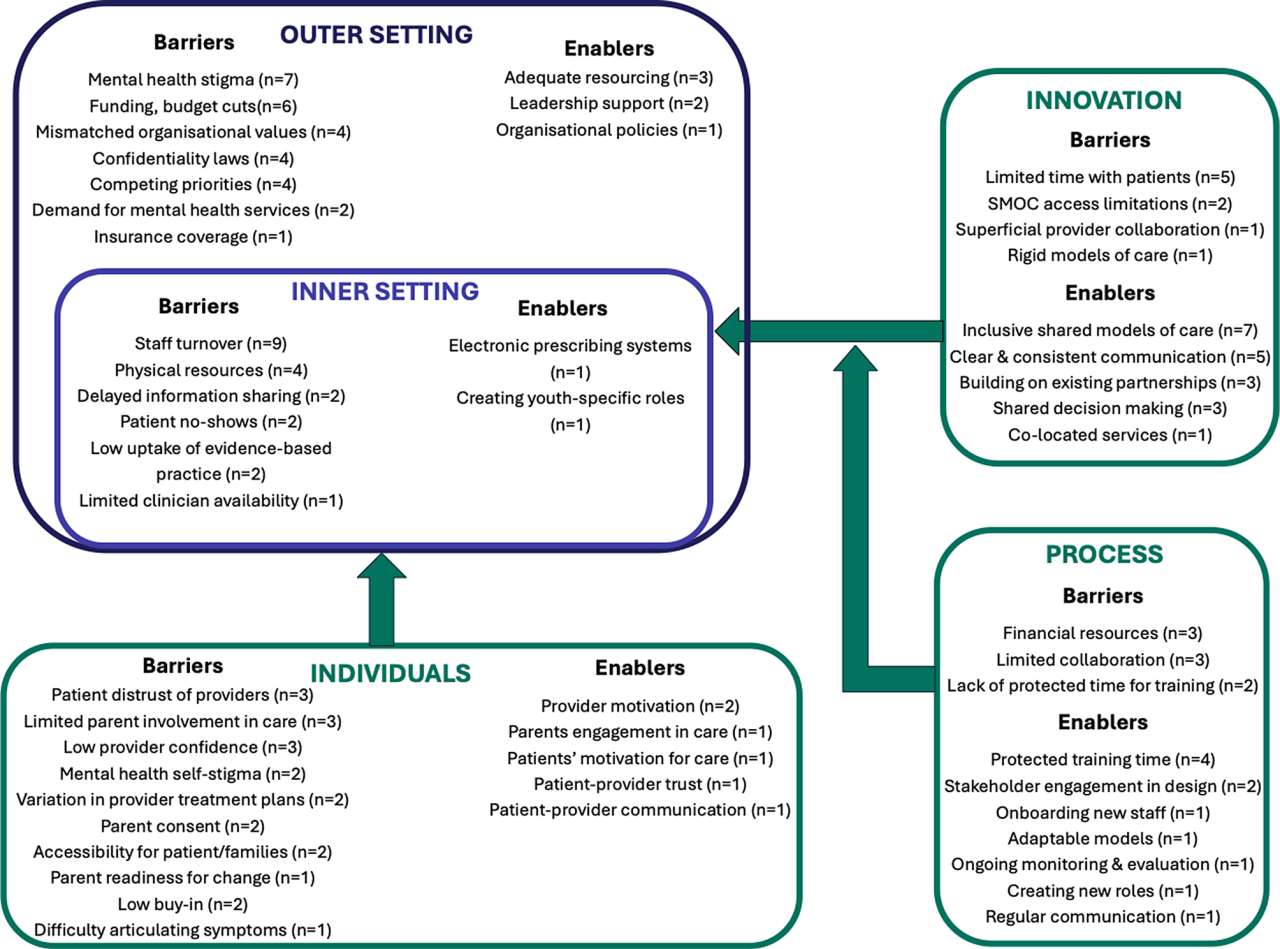
Summary of SMOC determinants mapped to CFIR domains

Summaries of the identified SMOC barriers, enablers, and implementation determinants are described below corresponding to the five CFIR domains (Fig. 3.)

### Characteristics of individuals domain

Barriers emerged within and across multiple stakeholder levels. The most common patient-level barrier was distrust of healthcare providers (*n* = 3) [47, 50, 57], with mental health self-stigma (*n* = 2) [45, 47], MHD impairing executive functioning [47] and difficulties articulating mental symptoms also proving problematic [44]. Parent-level barriers included limited engagement in their child’s care (*n* = 3) [44, 57, 62], services requiring parental consent (*n* = 2) [57, 62] and variations in parents’ readiness for change (*n* = 1) [44]. Structural barriers at both the patient and parent level included transportation and childcare costs to access services [50] and language and/or cultural barriers [57]. Barriers at the provider level included limited confidence among GPs to treat MHD (*n* = 3) [45, 47, 50], variability within and across healthcare providers’ treatment plans (*n* = 2) [45, 67], and limited buy-in to shared care (*n* = 2) [47].

Motivation was an enabler across all three groups. Young people’s willingness to engage in care was a facilitator [55], while some parents were proactive in seeking help for their child [44], and providers expressing a desire to improve care [44, 69]. Establishing trust and frequent communication between patients and providers was essential [47], as was valuing relationship-building between young people, parents and providers to improve health outcomes [65].

#### Innovation domain

 Barriers to SMOC included limited time with patients (e.g., need for longer appointments, extended clinic hours; *n* = 5) [44, 46, 47, 50, 59] and access limitations to SMOC (e.g., models limited to certain youth age groups; services available for up to 6 months; *n* = 2) [46, 49]. Rigid models of shared care (e.g., no walk-ins or same day services) and perceived superficial collaboration between providers [47] were additional barriers identified. These barriers resulted in duplicated workloads [49], confusion regarding provider’s responsibility for medication monitoring, patients’ eligibility for services, services available and referral pathways [44, 46, 47, 49, 51, 57, 59]. Two studies noted that young people themselves bridged these communication gaps between services [46, 47].

Conversely, clear communication across primary and secondary care was critical to SMOC [62, 65]. Four models discussed using ‘warm handoffs’ to facilitate this [54, 56, 59, 60]. A warm handoff is *“a transfer of care between two members of the healthcare team… [that] occurs in front of the patient and family… [allowing] patients and families to hear what is said and giving them the opportunity to clarify or correct information or ask questions about their care.“* [60, 71] Other modes of communication across care settings included electronic medical records (n = 4) [45, 54, 60, 68], telehealth platforms (n = 3) [53, 60, 64], real-time consultations (n = 3) [45, 56, 60], regular team meetings [49, 60], registry conferences [60, 67], telephone calls [54, 72] and progress notes [56].

Additionally, SMOC were supported when models were youth-specific, trauma-informed and/or inclusive of culture and diversity (*n* = 7) [44, 47, 48, 50, 51, 61, 64]. Other facilitators included building on existing clinical partnerships (*n* = 3) [48, 49, 51], reinforcing clinical decision making between healthcare providers (*n* = 3) [48, 49, 53] and co-located services which supported quick and direct clinician debriefs (*n* = 1) [56]. These features supported a clear scope of practice for providers [48], procedural consistency across sites [45, 59], reduced referral thresholds [45, 56, 59], improved medication management [53] and improved provider workloads [51].

### Inner setting domain

High staff turnover was a pervasive barrier (*n* = 9) [44–47, 50, 51, 54, 61, 62], along with limited physical resources (e.g., space, privacy; *n* = 4) [44, 47, 49, 57]. Delays in information sharing across settings (*n* = 2) [46, 51], patient no-shows (*n* = 2) [54, 59], low uptake of evidence-based practice (*n* = 2) [62, 67] were also identified, along with the isolated challenge of limited clinician availability noted in one study [59]. Enablers included settings using electronic prescribing systems [45] and creating youth-specific roles within the health systems (e.g., advisory councils, peer advocates) [50].

### Outer setting domain

Key barriers at this domain included the societal mental health stigma (*n* = 7) [44–47, 50, 57, 62], and funding constraints (*n* = 6) [44, 47, 50, 54, 57, 66]. Discrepancies between collaborating organisation expectations or philosophies were identified in four studies [48, 49, 51, 60], as were confidentiality laws preventing adequate information exchange (*n* = 4) [46, 47, 50, 57] and competing priorities in the health system (*n* = 4) [44, 46, 47, 50]. Excess demands for services (*n* = 2) [54, 57], and insurance coverage (e.g., reimbursement, termination of coverage) were other barriers to care [46]. Despite these barriers, identified enablers included adequate resourcing (*n* = 3) [47, 48, 66], strong leadership commitment (*n* = 2) [44, 55] and organisational policies supporting information exchange (*n* = 1) [48].

### Implementation process domain

Process barriers included limited financial sustainability (*n* = 3) [52, 54, 60], poor, or finite, collaboration across settings (*n* = 2) [44, 48, 52] and a lack of ongoing collaboration between stakeholders, and insufficient time for training or interagency work (*n* = 2) [44, 57]. Enablers included protecting time for providers’ upskilling or continuing education (*n* = 4) [52, 53, 60, 69] and designing models with local stakeholder consultations (*n* = 2) [59, 69]. Other enablers noted in one study each included updating onboarding for new staff to SMOC procedures [60], creating new roles (e.g., service coordinators) to support care provision across settings [69], regular team meetings to communicate and troubleshoot SMOC concerns [60] and model adaptability to local contexts [69]. Mautone et al. (2021) noted ongoing monitoring, evaluation and refinement were essential to SMOC implementation success [59].

## Discussion

We identified 25 shared models of care (SMOC) which integrated physical and/or sexual health with mental healthcare services for young people with mental health difficulties (MHD). While models often addressed physical health needs, sexual health inclusion was far less common. As seen in other reviews of integrated youth services, SMOC were variably described, inconsistently reported and implementation strategies were sparse [22, 32, 73]. CFIR proved useful for capturing implementation determinants of the often complex, cross-sectoral models we identified. Consistent with prior reviews using CFIR, identified barriers often arose at the inner and outer settings [74, 75]. Few models were designed or evaluated using theoretical frameworks, thereby limiting generalisability of findings. Future research should explore the scalability and sustainability of SMOC designed for young people with MHD beyond initial piloting, particularly in real-world, publicly funded health systems.

Included SMOC had limited focus on sexual health or wellbeing. Sexual health is key component to overall health and needs particular attention among adolescents and young adults as onset of sexual activity, and often high-risk sexual behaviours, occurs during these ages [76, 77]. SMOC often treated this domain as ancillary rather than a core component of care, reflecting broader health system silos and potentially reinforcing fragmented service experiences for young people [78, 79]. This mirrors findings from broader adolescent health research, where sensitive or stigmatised topics are often deprioritised [80]. We identified several provider-levels barriers, including confidence to address sexual or mental health concerns, insufficient training, and a lack of clear ownership, which can contribute to this pattern of avoidance in implementation [27]. Future work is needed to create inclusive, non-judgemental and confidential environments that reflect safety and belonging for young people to discuss sexuality, gender identity, and sexual wellbeing with their care providers. Australian SMOC that address co-occurring sexual and mental health needs provide an innovative example which could be replicated in other countries [81].

Consistent with other youth SMOC literature [82], we identified SMOC often included youth-friendly features facilitated engagement in care, including consideration for the young person’s gender identity, sexual orientation, cultural background and use of trauma-informed practices. There is a need for researchers and care providers to actively include young people in the design, delivery and evaluation of SMOC; however young people are rarely included in the co-design or evaluation of health services research [83]. Input from young people with MHD is vital to ensure the services designed are relevant, accessible, non-stigmatising and inclusive of their needs, particularly for those from marginalised communities. Prioritising meaningful engagement with young people at the outset of SMOC development is required from researchers, service providers and decision makers to support adolescent-responsive health systems, while testing the effectiveness of these SMOC can contribute evidence to the clinical outcomes associated with this approach to care [84–86].

SMOC need to ensure care components and services are adaptable to the needs of young people with MHD, yet can be tailored to address the unique needs of individual patients. Young people aged 10–25 experience a range of developmental and life milestones during this time, and SMOC need to account for the variations in care needs within and across these ages (e.g., transitions to new work or school environments and/or into adult healthcare services.) Many innovative SMOC designed for children and adolescents do not sustain patient support as they transition into adult care [87, 88]. This transition in care is associated with year-long care gaps, reduced treatment adherence, and poorer outcomes among young people [87, 89, 90]. SMOC should account for these transitions and potential fluctuations in engagement from patients as they navigate complex life milestones. We identified flexible scheduling, warm handovers, and care coordinators as facilitators to SMOC [44, 54, 56, 59, 60], which should be considered when designing care models for this population. Future research should also examine how physical, sexual, and mental health needs evolve from adolescence into young adulthood to identify earlier opportunities for health service responses.

Clear communication protocols are vital to SMOC [91]. We identified electronic records, telehealth, and warm handoffs as communication channels which helped enable shared care in our review. However, delays in information sharing across settings and confidentiality laws could hinder communication, resulting in duplicated workloads and confusion regarding division of clinical responsibilities [92]. Clarifying clinician responsibilities across disciplines, supported by interprofessional education and joint supervision structures, is essential to strengthen shared accountability and reduce fragmentation. While digital platforms can support this, our findings suggest they should supplement, not replace, personal trust and relationships, as overreliance on technology risks furthering the sense of superficial collaboration among providers [93].

Mental health stigma remains a pervasive societal issue [94, 95], despite numerous policies and interventions aimed at addressing it [2]. Several SMOC were delivered through school and community settings, yet these sectors are often overlooked in health service planning and implementation. This highlights an opportunity for cross-sector strategies that embed shared care within young people’s daily environments, which can reduce accessibility barriers and normalise help-seeking behaviours [96]. Sustained public health efforts and upstream investment remain vital to address the root causes of mental health stigma across cultures and generations.

For policy makers, these findings highlight the importance of embedding shared care frameworks within national adolescent and mental health strategies, ensuring parity of physical, sexual, and mental health needs. For practitioners, investing in cross-sector communication mechanisms, structured referral protocols, and joint training can strengthen implementation fidelity and improve continuity of care. Commissioners and service planners can use these results to guide resource allocation and co-design efforts for sustainable, youth-centred service models. Applying CFIR in scoping reviews of youth health integration provides a transferable model for identifying context-sensitive implementation strategies across health systems. This approach demonstrates the value of theory-informed synthesis to bridge gaps between research and service delivery, supporting implementation planning across diverse settings.

### Strengths & limitations

Strengths of this review include the use of JBI methodology, adherence to PRISMA-ScR reporting [36, 37], optional critical appraisals with MMAT, and application of CFIR to provide theoretical and methodological rigour [26, 42]. Limitations include variability in shared care terminology, underreporting of implementation strategies, and the concentration of studies in high-income countries, particularly the US and Australia. Findings may therefore may not be generalisable to lower-resource settings, underscoring the need for research across more diverse health systems.

## Conclusion

This review identified 25 SMOC integrating mental with physical and sexual health care for young people with MHD. While most models incorporated referral, assessment, treatment, and links to external services, sexual health remained under-addressed. Ensuring parity between physical, sexual, and mental health is essential, yet implementation continues to be limited by individual and contextual factors. Deliberate health services planning is needed between young people, providers and researchers to co-design youth-specific, cross-sector models that embed communication protocols and staff training to support the delivery of holistic, accessible care for young people with MHD.

## Supplementary Information

Below is the link to the electronic supplementary material.

Supplementary Material 1

Supplementary Material 2

## Data Availability

The datasets used and/or analysed during the current study are available from the corresponding author on reasonable request.

## Abbreviations

BMI: Body mass index
CFIR: Consolidated Framework for Implementation Research
ICD-11: International Classification of Diseases- 11th Revision
GP: General practitioner
MMAT: Mixed Methods Appraisal Tool
MHD: Mental health difficulty
PCC: Population–Concept–Context
PRISMA: Preferred Reporting Items for Systematic Reviews and Meta-Analyses
ScR: Scoping Review
SMOC: Shared model of care
STI: Sexually Transmitted Infection
US: United States
WHO: World health organisation
