# Supplementary material for: Implementing shared care models for young people with mental health difficulties: a consolidated framework for implementation research- informed scoping review of service integration across physical, sexual and mental health domains
Source: BMC Health Serv Res. 2026 Feb 20;26:415. doi: 10.1186/s12913-026-14178-x (PMC13032585; doi:10.1186/s12913-026-14178-x)
Supplement: Supplementary file 1 — Supplementary Material 1 [file 12913_2026_14178_MOESM1_ESM.docx]

**Additional File 1.** Preliminary Search Strategy

**Database: Web of Science Core Collection**

**Search date: 21^st^ October, 2024**

**Yield: 1436 citations.**  
 

(barrier* OR determinant* OR enable* OR facilitat* OR implement*)

AND

(anxiet* OR anxious OR "conduct disorder*" OR depress* OR "eating disorder*" OR ((mental*) NEAR/2 (disorder* OR health* OR ill*)) OR psychiatric OR psychosis OR psychotic OR schizophreni* OR "self harm*" OR suicid*)

AND

("body mass" OR cardio* OR chronic OR comorbidit* OR "co morbidit*" OR contracepti* OR diabet* OR ((family OR general OR primary) NEAR/2 (care OR doctor* OR physician* OR practi*)) OR "GP" OR "HIV" OR ((impulsiv* OR risk*) NEAR/2  (behaviour* OR behavior*)) OR obes* OR "physical health" OR pregnan* OR "risk taking" OR "sexual health" OR "STD*" OR "STI" OR "unsafe sex" OR (weight NEAR/2 (gain OR loss)))

AND

((("co-ordinat*" OR coordinat* OR "co-locat*" OR colocat* OR collaborat* OR integrat* OR shared*) NEAR/2 (care OR decision* OR health* OR intervention* OR model* OR program*)))

AND

(adolescen* OR boy* OR child* OR girl* OR juvenile* OR learner* OR minor* OR pediatric* OR paediatric* OR pupil* OR schoolboy* OR schoolgirl* OR (secondary NEAR/2 (education OR school*)) OR student* OR teen* OR young* OR youth*)
