# Supplementary material for: Implementing shared care models for young people with mental health difficulties: a consolidated framework for implementation research- informed scoping review of service integration across physical, sexual and mental health domains
Source: BMC Health Serv Res. 2026 Feb 20;26:415. doi: 10.1186/s12913-026-14178-x (PMC13032585; doi:10.1186/s12913-026-14178-x)
Supplement: Supplementary file 2 — Supplementary Material 2 [file 12913_2026_14178_MOESM2_ESM.docx]

Additional File 3. Summary of MMAT Scoring Details ^1^

| **Lead author & year** | **Responses to Criteria from the Mixed Methods Appraisal Tool** | | | | | **Total MMAT Score (out of 5 stars *****)** |
| --- | --- | --- | --- | --- | --- | --- |
| **Qualitative Studies** | **1.1. Is the qualitative approach appropriate to answer the research question?** | **1.2. Are the qualitative data collection methods adequate to address the research question?** | **1.3. Are the findings adequately derived from the data?** | **1.4. Is the interpretation of results sufficiently substantiated by data?** | **1.5. Is there coherence between qualitative data sources, collection, analysis and interpretation?** |  |
| Katz-Wise et al., 2020 | Yes | Yes | Yes | Yes | Yes | 5/5 |
| Hugunin et al., 2023 | Yes | Yes | Yes | Yes | Yes | 5/5 |
| Lai et al., 2016 | Yes | Yes | Yes | Yes | Yes | 5/5 |
| Morgan et al., 2018 | Yes | Yes | Yes | Yes | Yes | 5/5 |
| Nash et al., 2020 | Yes | Yes | Yes | Yes | Yes | 5/5 |
| Rooney et al., 2021 | Yes | Yes | Can’t tell | No | Yes | 3/5 |
| Rodriguez et al., 2019 | Yes | Yes | Yes | Yes | Yes | 5/5 |
| Rungan et al., 2024a | Yes | Can’t tell | Yes | Yes | Yes | 4/5 |
| Hacker et al., 2013 | Yes | Yes | Yes | Yes | Yes | 5/5 |
|  | | | | | | |
| **Quantitative Descriptive Studies** | **4.1. Is the sampling strategy relevant to address the research question?** | **4.2. Is the sample representative of the target population?** | **4.3. Are the measurements appropriate?** | **4.4. Is the risk of nonresponse bias low?** | **4.5. Is the statistical analysis appropriate to answer the research question?** |  |
| Adams et al., 2016 | Yes | Yes | Yes | Can’t tell | Yes | 4/5 |
| White et al., 2021 | Yes | Yes | Yes | Can’t tell | Yes | 4/5 |
| Brunette et al., 2023 | Yes | Yes | Yes | Can’t tell | Yes | 4/5 |
| Hine et al., 2017 | Yes | Yes | Yes | No | Yes | 4/5 |
| Liu et al., 2010 | Yes | Yes | Yes | Can’t tell | Yes | 4/5 |
| Mathias et al., 2022 | Yes | Yes | Yes | Can’t tell | Yes | 4/5 |
| Walter et al., 2018 | Yes | Yes | Yes | Yes | Yes | 5/5 |
| Schweitzer et al., 2023 | Yes | Yes | Yes | Can’t tell | Can’t tell | 3/5 |
| Mautone et al., 2021 | Yes | Yes | Yes | Can’t tell | Yes | 4/5 |
